# Development of new screening tools to evaluate dog exposure to *Phlebotomus tobbi* and *Phlebotomus papatasi* sand flies

**DOI:** 10.1186/s13071-026-07286-4

**Published:** 2026-02-27

**Authors:** Iva Kolářová, Kristýna Jelínková, Helena Přibylová, Suha K. Arserim, Metin Pekagirbas, Kardelen Yetismis, Umut Berberoglu, Unal Altug, Yusuf Özbel, Seray Töz, Petr Volf, Carla Maia

**Affiliations:** 1 Department of Parasitology, Faculty of Science, Charles University, Prague, Czech Republic; 2 Vocational School of Health Sciences, Celal Bayar University, Manisa, Turkey; 3 Department of Parasitology, Veterinary School, Aydin Adnan Menderes University, Aydin, Turkey; 4 Department of Parasitology, Faculty of Medicine, Ege University, Izmir, Turkey; 5 General Directorate of Public Health, Turkish Ministry of Health, Ankara, Turkey; 6 Present Address: Global Health and Tropical Medicine (GHTM), LA-REAL , Global Health and Tropical Medicine (GHTM), LA-, Universidade NOVA de Lisboa, Lisbon, Portugal

**Keywords:** *Phlebotomus papatasi*, *Phlebotomus tobbi*, Yellow-related protein, Apyrase, D7 protein, Dog, Serology, ELISA, Leishmaniasis control, Marker of exposure

## Abstract

**Background:**

Leishmaniases are a group of medically and veterinary important diseases caused by protozoan parasites of the genus *Leishmania* (Kinetoplastida) and transmitted by blood-feeding female sand flies (Diptera: Phlebotominae). To assess the risk of *Leishmania* transmission, host exposure to sand fly bites can be measured through the detection of host antibodies to vector salivary proteins. Anti-sand fly saliva antibodies are elicited by repeated exposure of the mammalian host to sand fly salivary proteins deposited into the host skin during blood feeding. These antibodies are species-specific and correlate with the intensity of host exposure to sand fly bites. The aim of our study was to develop enzyme-linked immunosorbent assays (ELISAs) on the basis of recombinant sand fly salivary antigens as tools to measure exposure to sand fly bites, hence serving as risk markers for *Leishmania* transmission. We focused on two Old World vector sand fly species: *Phlebotomus tobbi* as a vector of *Leishmania infantum*, and *Phlebotomus papatasi* as a vector of *Leishmania major*.

**Methods:**

Dog sera from endemic areas in Türkiye were used to characterise the main salivary antigens of *P. papatasi* and *P. tobbi* in immunoprecipitation and immunoblot assays, followed by proteomic analysis. Four candidate salivary proteins from each species were expressed in *Escherichia coli* and subsequently evaluated and validated in an ELISA as potential risk markers of dog exposure to sand flies.

**Results:**

Among the eight tested recombinant candidates, *P. tobbi* rSP38 (a yellow-related protein), *P. papatasi* rSP36 (an apyrase) and *P. papatasi* rSP42 (a yellow-related protein) were identified as the most reliable antigens to replace salivary gland homogenate (SGH) in serological assays. They demonstrated high correlation with SGH and exhibited high sensitivity and specificity.

**Conclusions:**

These recombinant antigens can be developed into a standardized assay to measure dog exposure to sand flies, which can serve as a complementing tool for leishmaniasis surveillance and control.

**Graphical Abstract:**

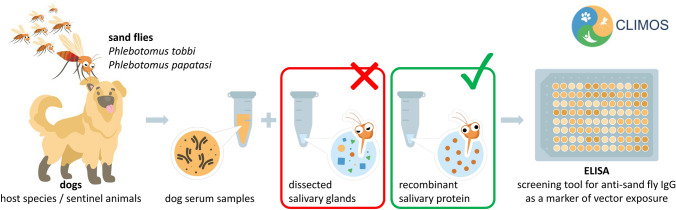

**Supplementary Information:**

The online version contains supplementary material available at 10.1186/s13071-026-07286-4.

## Background

Sand flies are bloodsucking insects (Diptera: Phlebotominae) and important vectors of human and animal pathogens causing so-called sand fly-borne (SFB) diseases such as Pappataci fever, Carrión’s disease and leishmaniases [1]. The latter are endemic mainly in the tropics and subtropics but also occur in warmer temperate regions. In humans, leishmaniases have three basic clinical forms, ranging from self-healing cutaneous leishmaniasis (CL) to disfiguring mucocutaneous (MCL) and life-threatening visceral leishmaniasis (VL). An estimated 12 million people worldwide are infected with *Leishmania* parasites, resulting in 20–40,000 deaths annually mainly due to VL [2, 3]. Dogs serve as important reservoir hosts for human VL, also possessing a wide range of clinical signs themselves, from being asymptomatic to a chronic viscero-cutaneous disease, which is lethal if left untreated. Canine leishmaniasis (CanL) seroprevalence is estimated at around 16% globally [4], reaching up to 36% in certain endemic regions [5, 6]. Prevention involves repellent and insecticide-based products, and the only vaccine against leishmaniasis is available in Europe for dogs and does not prevent infection [7, 8].

The epidemiological situation of leishmaniases is dynamic, influenced by a number of factors that pose a risk of newly emerging or re-emerging foci and spread to surrounding non-endemic countries. These risk factors include movement of infected humans and animals, and climate and environmental changes [1, 9]. The situation can be monitored in several ways, such as regular sand fly trapping with subsequent species identification, blood meal analysis and screening for SFB pathogens [10–12]. A complementary tool is available to monitor individual host exposure to sand flies. This tool can assess host exposure to sand fly bites by using serological tests that detect specific antibodies against sand fly saliva. These antibodies are elicited by repeated bites, as sand flies deposit saliva into the host’s skin to facilitate blood feeding by inhibiting haemostatic reactions (inflammation, vasoconstriction, coagulation, platelet aggregation and degranulation). At the same time, saliva also contains antigenic proteins that trigger the production of sand fly-specific antibodies. The presence and amount of these antibodies then indicate sand fly activity in a given area and, consequently, can serve as risk markers for transmission of the SFB pathogens [13].

Antibodies to sand fly saliva can be measured by using sand fly salivary glands that have been manually dissected from sand fly females [14]; however, recombinant proteins offer a more standardised and cost-effective approach independent of maintaining colonised sand flies and manual dissection. The use of recombinant salivary antigens has been validated in several studies (reviewed e.g. [13, 15]). In dogs, they have been developed and used to measure anti-saliva antibodies against the New World sand fly *Lutzomyia longipalpis* [16–18], and two Old World sand flies – *Phlebotomus orientalis* [19, 20] and *Phlebotomus perniciosus* [21, 22]. The serology assay based on the *P. perniciosus* recombinant salivary protein SP03B is the most advanced, it has been validated in a large variety of epidemiological settings [23–28], and is also available as a rapid test [29–31].

This study aimed to develop new recombinants to monitor dog exposure to bites of two other important vector species – *Phlebotomus tobbi* and *Phlebotomus papatasi*. *Phlebotomus tobbi* is distributed mainly in the eastern Mediterranean and Middle East [32–34] and transmits *Leishmania infantum*, causing primarily human VL and CanL [32, 35]. *Phlebotomus papatasi* has a wide distribution spanning the western Mediterranean (including North Africa) and extending eastward through the Middle East into Central Asia and parts of the Indian subcontinent; it is a proven vector of *Leishmania major*, causing human CL [32–34]. Both vectors are opportunistic and have been shown to blood feed on humans as well as on dogs [11, 35, 36]. The presence of dogs in close proximity to human habitations thus allows for their use as sentinel hosts in epidemiological studies that address the efficacy of protective measures and risk of infection also for humans.

## Methods

### Dog sera

Canine serum samples were collected between November 2023 and January 2024 (*n* = 201), in Adana (Kozan town, Koyunevi village and surrounding villages) and Manisa (Alasehir town, Ucpinar village) provinces in Türkiye, in areas where *P. tobbi* or *P. papatasi* are endemic, respectively [37–39]. Negative control serum samples originated from the Czech Republic, a sand fly-free area, kindly provided by MVDr. Kamil Sedlák, director of the State Veterinary Institute Prague (*n* = 41). Serum samples were stored at −80 °C and their aliquots at −20 °C.

### Sand fly salivary gland homogenate

Laboratory colonies of *P. papatasi* and *P. tobbi* (established in 2005 and 2008, respectively) originated from Türkiye and were maintained in the insectary at the Department of Parasitology, Charles University, Czech Republic, under standard conditions [40, 41]. Salivary glands were dissected from 4–6-day-old female sand flies into 0.9% NaCl (20 glands per 20 μl of physiological solution) and stored at −20 °C (or at −80 °C for longer storage). Before use, salivary glands were disrupted by three freeze–thaw cycles in liquid nitrogen to obtain the salivary gland homogenate (SGH).

### Enzyme-linked immunosorbent assay (ELISA)

ELISA plates (Thermo Scientific, cat. no. 478042) were coated with *P. papatasi* or *P. tobbi* SGH (corresponding to 0.2 gland per well) diluted in 20 mM carbonate–bicarbonate buffer (pH 9.6) at 4 °C overnight. After washing with PBS-Tw (phosphate-buffered saline,  0.05% Tween 20), the plates were blocked with 6% non-fat dried milk (ITW Reagents, cat. no. A0830) diluted in PBS-Tw and incubated for 60 min at 37 °C. After another washing, the plates were incubated with sera diluted 1:100 in 2% non-fat dried milk for 90 min at 37 °C, then washed and incubated for 60 min at 37 °C with peroxidase-conjugated anti-dog IgG (Bethyl Laboratories, cat. no. A40-123P) diluted 1:9,000 in PBS-Tw. The chromogenic reaction was developed in McIlwain phosphate–citrate buffer (pH 5.5) with orthophenylendiamine (OPD; Sigma-Aldrich, cat. no. P8287) and hydrogen peroxide (Fluka, cat. no. 95321) in the dark. The reaction was stopped by adding 10% sulfuric acid, and the absorbance was measured at 492 nm using a Tecan Infinite M200 microplate reader. Each serum sample was run in duplicate. This protocol was used to generate data for Fig. 1.

ELISA that was performed with recombinant proteins as the antigen was modified to increase the assay sensitivity as follows: plates (Thermo Scientific, cat. no. 3855) were coated with recombinant protein (0.2 μg/well) or SGH (equivalent to 0.1 gland per well) for 2 h at room temperature (RT), blocked, and incubated with sera diluted 1:300 overnight at 4 °C. The next day, the plates were washed and incubated with anti-dog IgG secondary antibody diluted 1:40,000 in PBS-Tw. The chromogenic reaction was developed in 3,3′,5,5′-tetramethylbenzidine substrate solution (TMB; Sigma-Aldrich, cat. no. T4444) in the dark for 10 min. The absorbance was measured at 450 nm. This optimised protocol was used to generate data for Figs. 3 and 4 and Tables 4 and 5.

### Electrophoresis and immunoblot

The reactivity of anti-*P. tobbi* and anti-*P. papatasi* saliva IgG with SGH was also tested on immunoblot. SGH was mixed 1:1 with non-reducing Laemli Sample Buffer (BioRad, cat. no. 161–0737), boiled for 3 min and loaded on a 10% polyacrylamide gel in an equivalent of 1.5 salivary gland per millimeter of the well width (i.e. 20 glands per well of the 5-well comb). SGH was separated along with the Precision Plus Protein Dual Color Standards (BioRad, cat. no. 1610374) using a Mini-PROTEAN apparatus (Bio-Rad) under limited voltage (200 V). Separated proteins were either stained by Coomassie Blue (BioRad, cat. no. 1610786) or transferred onto a nitrocellulose membrane using the iBLOT system (Invitrogen). The membrane was cut into strips and blocked with 6% non-fat dried milk (ITW Reagents, cat. no. A0830) diluted in PBS-Tw overnight at 4 °C. After washing (PBS-Tw), the strips were incubated with sera diluted 1:50 in PBS-Tw for 60 min at RT. After another washing, peroxidase-conjugated anti-dog IgG (Bethyl Laboratories, cat. no. A40-123P) diluted 1:1000 in PBS-Tw was added for 60 min at RT. The chromogenic reaction was developed in a substrate solution containing diaminobenzidine (DAB; MP, cat. no. 980681) and hydrogen peroxide (Fluka, cat. no. 95321). The reactivity of recombinant salivary proteins was tested using the same protocol by separating 6 µg of the recombinant per well of the 5-well comb, i.e. 0.5 µg/mm of the well width.

### Immunoprecipitation assay

Antigenic proteins were characterised using an immunoprecipitation assay kit (Invitrogen, cat. no. 10007D) following manufacturer protocol no. MAN0017348, with some modifications. First, beads were washed and collected using the magnet. A pool of three dog serum samples (10 µl each in a 200 µl Ab binding and washing buffer), was added to 1.5 mg of magnetic beads and incubated with rotation for 10 min at RT. Beads were incubated either with a pool of sera highly positive for anti-*P. papatasi* SGH IgG or highly positive for anti-*P. tobbi* SGH IgG. Only sera that were highly positive for the target species and negative for the other one were used to ensure species-specificity of the selected antigens. Sera negative for both anti-*P. papatasi* and anti-*P. tobbi* SGH IgG served as controls. Unbound antibodies were washed out, and antibody-conjugated beads were resuspended in 300 µl of Tris–NaCl (25 mM Tris, 150 mM NaCl, pH 7.9), containing either 20 salivary glands of *P. papatasi* or 30 salivary glands of *P. tobbi,* and incubated while rolling for 150 min at 4 °C. After incubation, unbound salivary proteins were washed out, and beads were transferred to a clean tube, resuspended in 20 µl of elution buffer and incubated while rolling for 2 min at RT. Eluted protein samples were kept at −80 °C until proteomic analysis.

### Proteomic analysis

Eluted samples were firstly prepared for protein digestion by incubation in 100 mM triethylammonium bicarbonate buffer(TEAB; Thermo Scientific, cat. no. 90114), 2% sodium deoxycholate (SDS; Sigma-Aldrich, cat. no. 30970), 40 mM chloroacetamide, and 10 mM TCEP HCl (tris(2-carboxyethyl) phosphine hydrochloride; Thermo Scientific, cat. no. 20491). Samples were denatured by incubation at 95 °C for 5 min and further processed using SP3 beads (Sigma-Aldrich, cat. no. GE45152105050250) according to [42]. Briefly, proteins were allowed to bind to 5 µl of SP3 beads in lysis buffer containing 60% ethanol for 5 min at RT. The unbound supernatant was discarded, and beads were washed with 180 ul of 80% acetonitrile (ACN), followed by two washings in 180 µl of 80% ethanol. After washing, samples were digested with 1 µg of trypsine (Thermo Fisher, cat. no. 90057) at 37 °C overnight. Afterward, digested samples were acidified with 1% trifluoroacetic acid (TFA; Thermo Scientific, cat. no. 28901), and peptides were desalted using in-house-made stage tips packed with C18 disks (Empore) according to [43]. The liquid chromatography–mass spectrometry (LC–MS) analysis was performed using nano reversed-phase columns (EASY-Spray column, 50 cm × 75 um ID, PepMap C18, 2 um particles, 100 Å pore size; Thermo Scientific, cat. no. 03–255-018), mobile phase buffer A (0.1% formic acid) and mobile phase B (acetonitrile, 0.1% formic acid). Samples were loaded onto the trap column (C18 PepMap100, 5 um particle size, 300 um × 5 mm; Thermo Scientific) for 4 min at 18 µl/min in loading buffer (2% acetonitrile, 0.1% trifluoroacetic acid). Peptides were eluted with mobile phase B gradient from 4% to 35% B for 60 min. Eluting peptide cations were converted to gas-phase ions by electrospray ionization and analysed on a Thermo Orbitrap Fusion mass analyser (Q-OT-qIT, Thermo Scientific). Survey scans of peptide precursors from 350 to 1400 *m*/*z* were performed in Orbitrap at 120 K resolution (at 200 *m*/*z*) with a 5 × 10^5^ ion count target. Tandem MS was performed by isolation at 1.5 Th with the quadrupole, higher-energy collisional dissociation fragmentation with normalized collision energy of 30 and rapid scan MS analysis in the ion trap. The MS2 ion count target was set to 10^4^, and the max injection time was 35 ms. Only those precursors with charge state 2–6 were sampled for MS2. The dynamic exclusion duration was set to 45 s with a 10-ppm tolerance around the selected precursor and its isotopes. Monoisotopic precursor selection was turned on. The instrument was run in top speed mode with 2 s cycles [44]. All data were analysed and quantified with the MaxQuant software (version 2.0.3.0) [45]. The false discovery rate (FDR) was set to 1% for both proteins and peptides and a minimum peptide length to seven amino acids. The Andromeda search engine was used for the tandem mass spectrometry (MS/MS) spectra search against the *Canis lupus familiaris* database (downloaded from uniprot.org, OPPG version), and the National Center for Biotechnology Information (NCBI) databases for *P. tobbi* (225 entries) and *P. papatasi* (21,690 entries). Enzyme specificity was set as C-terminal to Arg and Lys, also allowing cleavage at proline bonds and a maximum of two missed cleavages. Carbamidomethylation of cysteine was selected as fixed and N-terminal protein acetylation and methionine oxidation as variable modifications. Quantifications were performed with the label-free algorithm in MaxQuant [45]. Data analysis was performed using Perseus 1.6.15.0 software [46]. LC–MS and data analyses were performed in the Laboratory of Mass Spectrometry at the Biotechnology and Biomedicine Centre of the Academy of Sciences and Charles University (BIOCEV, Czech Republic).

### Bioinformatic analyses

The following bioinformatic tools were used to characterize sequences of selected candidate proteins: SignalP-5.0 [47], NetNGlyc-1.0 [48], CD-Search [49] and ScanProsite [50].

### Recombinant proteins production

Sand fly salivary proteins (four of each species) were expressed in a bacterial expression system (Additional File 1: Supplementary Table S1). Each protein coding sequence without a signal peptide part (identified by using SignalP-5.0 [47]) was synthesised as codon optimised for *Escherichia coli* and cloned into the modified pET-28aN(+)-TEV vector between Ncol and Xhol sites (GenScript). Expression and solubility tests, expressions in 1 L of Luria–Bertani (LB) media, and purifications were performed at the Core Facility Protein Production (The Centre of Molecular Structure, Institute of Biotechnology, Czech Academy of Sciences).

Recombinant TOB-rSP38, TOB-rSP56, TOB-rSP60, PAP-rSP36, and PAP-rSP42 proteins were expressed in the *E. coli* BL21(DE3) cells (New England Biolabs) as N-terminal 6× His-tagged proteins. Cells were cultivated at 37 °C until they reached optical density of approximately 0.6, then the temperature was lowered, and protein expression was induced by 0.5 mM isopropyl β-d-1-thiogalactopyranoside (IPTG) and continued for 18 h at 20 °C. Cells were harvested by centrifugation at 5000*g* for 15 min at 4 °C and stored at –21 °C. Target proteins were insoluble and purified under denaturing conditions in 8 M urea. Cells were lysed by sonication in lysis buffer (buffer A = 50 mM Tris–HCl pH 8, 300 mM NaCl, with approximately 125 U of benzonase nuclease HC, Millipore) and spun at 40,000*g* for 30 min at 4 °C. The pellet was resuspended in 150–180 mL of denaturing buffer (buffer B = 50 mM Tris–HCl pH 8, 300 mM NaCl, 8 M urea, with 1 mM imidazole) using a glass homogenizer, incubated for 60 min at RT with rotation, sonicated and centrifuged at 40,000*g* for 30 min at 18 °C. The supernatant was incubated with 2 mL of PureCube 100 Ni-INDIGO agarose resin (Cube Biotech) equilibrated with buffer B for 30 min at RT and run through the gravity-flow column, washed (buffer B: 10 CV) and eluted (buffer B with 150 mM imidazole). Fractions were screened using SDS–PAGE, and subsequently positive fractions were pooled and dialysed against 1 L of buffer B without imidazole in SnakeSkin™ Dialysis Tubing (Thermo Scientific) overnight at 10 °C, followed by a concentration step using VivaSpin 10 kDa-HY (Sartorius). Each protein sample was aliquoted, snap-frozen in liquid nitrogen and stored at –80 °C.

Recombinant PAP-rSP40 protein was expressed in the same way as described above with the following modifications: it was expressed as C-terminal 6× His-tagged fusion proteins, and the putative active proteolytic site (H172-D222-S316) was mutated (S316A).

Recombinant TOB-rSP10 and PAP-rSP32 proteins were expressed in the same way as described above (N-terminal and C-terminal 6× His-tagged, respectively). However, the target proteins were soluble and thus purified as follows. The pellet was resuspended in lysis buffer A (10 mM Tris pH 8, 500 mM NaCl) with a cOmpete mini tablet (Roche) and with approximately 125 U of benzonase nuclease HC (Millipore), lysed by sonication and centrifuged at 40,000*g* for 30 min at 4 °C. The supernatant was purified through the gravity-flow column with 1 mL of PureCube 100 Ni-INDIGO agarose resin (Cube Biotech), equilibrated with buffer C (50 mM Tris–HCl, 150 mM NaCl, pH 7.5 for TOB-rSP10; 20 mM NaH_2_PO_4_, 20 mM Na_2_HPO_4_, 500 mM NaCl, pH 7.4 for PAP-rSP32), washed and eluted with buffer C containing additional 300 mM and 50 mM imidazole, respectively. Eluted fractions of TOB-rSP10 were pooled together and dialysed in buffer C overnight at 10 °C. Pooled fractions of PAP-rSP32 protein were cleaved by 300 µg of tobacco etch virus protease (TEV; to remove the affinity tag, PAP-rSP32) in dialysis, overnight at 10 °C. Cleaved PAP-rSP32 was purified using reverse nickel chelating chromatography on HisCube Ni-INDIGO column (the sample was run twice through the column at 10 °C in buffer C), and fractions containing the protein were pooled and concentrated using VivaSpin 10 kDa-HY (Sartorius). The final purification step for both TOB-rSP10 and PAP-rSP32 was size-exclusion chromatography on HiLoad Superdex 200 16/600 column (Cytiva) attached to an NGC Chromatography System (Bio-Rad), at 10 °C, in 1× PBS. Final fractions were analysed using SDS–PAGE (Additional File 2: Supplementary Fig. S1), concentrated, aliquoted, snap-frozen in liquid nitrogen and stored at –80 °C.

Protein concentrations were estimated by DS-11PLUS Microvolume Spectrophotometer DeNovix at 280 nm and calculated using the extinction coefficients. Identities of all proteins were verified by mass spectrometry.

### Statistical analysis

Statistical analysis was performed using GraphPad Prism version 8.0.1. In ELISA, the samples were tested in duplicate, and the average values were used for downstream analyses. The difference between Turkish sera and Czech negative controls was evaluated using the non-parametric Mann Whitney *U* test for differences in medians. The non-parametric Spearman rank correlation test was used to assess correlations between anti-SGH and anti-recombinant protein IgG antibody levels. The cut-off values were calculated as the mean of the negative control samples plus 3 standard deviations. The cut-off value was used to calculate sensitivity, specificity, negative predictive value (NPV) and positive predictive value (PPV) parameters to assess the performance of the recombinant proteins in comparison with SGH as the antigen in ELISA tests. A *P*-value < 0.05 was considered to be statistically significant.

## Results

**Fig. 1:**
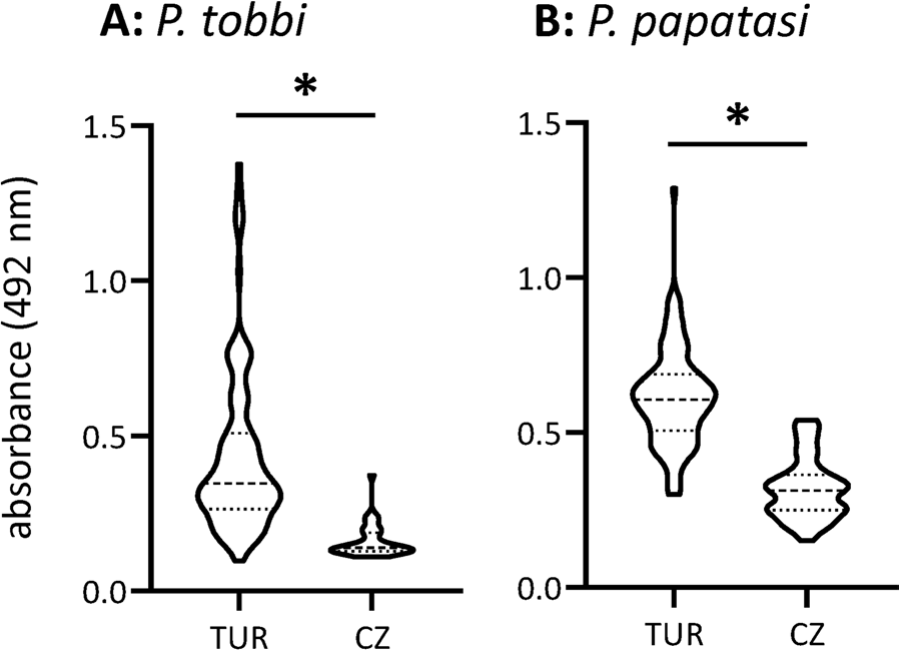
ELISA screening for anti-*Phlebotomus tobbi* and anti-*P. papatasi* SGH IgG antibodies. Sera were collected in Türkiye (TUR; *n* = 100) in *P. tobbi*- or *P. papatasi*-endemic areas and compared with negative control samples collected in the Czech Republic (CZ; *n* = 38), a sand fly-free area. The data are presented as a violin plot, with the dashed line marking the median value and the two dotted lines representing Q1 and Q3 quartiles. An asterisk (*) indicates significant differences (*P* < 0.0001) between Turkish sera and negative Czech controls analysed by a non-parametric two-tailed Mann Whitney test

### Anti-sand fly saliva IgG antibodies

Dog sera collected in *P. tobbi*- or *P. papatasi*-endemic areas were screened for IgG antibodies against sand fly saliva using salivary gland homogenate (SGH) as an antigen (Fig. 1). Serum samples from Türkiye had significantly higher levels of IgG antibodies against *P. tobbi* as well as against *P. papatasi* SGH than the controls from the Czech Republic, the non-endemic country (*P* < 0.0001).

Reactivity of sera highly positive by ELISA was further confirmed by immunoblot. In *P. tobbi*, selected serum samples showed a uniform pattern, strongly recognizing several antigenic bands, while *P. papatasi* serum samples showed more diverse patterns (data not shown).

### Selection of salivary antigens for recombinant production

**Fig. 2:**
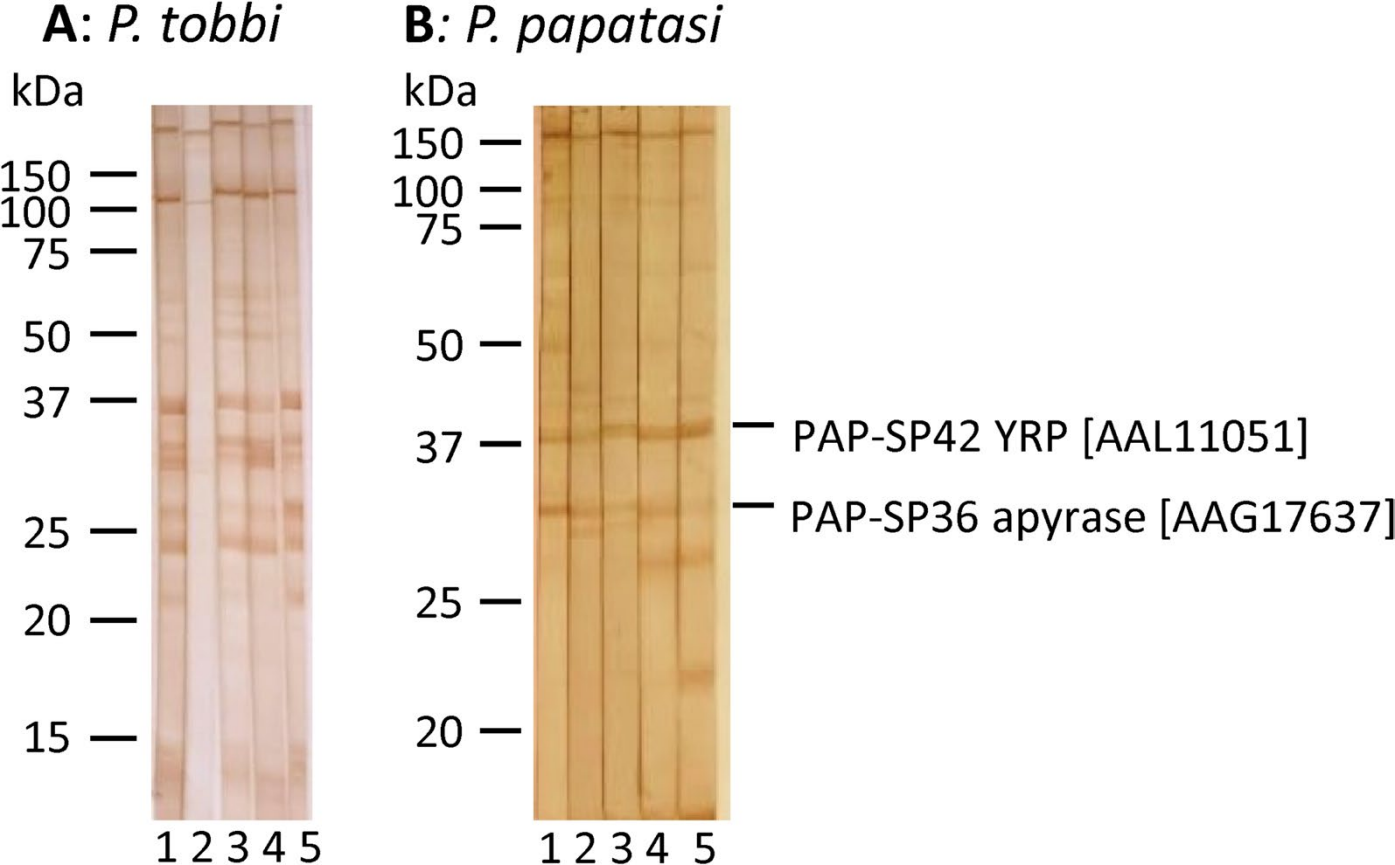
Reactivity of anti-salivary glands homogenate IgG on immunoblot. Salivary gland homogenates of *Phlebotomus tobbi* (**A**) and *Phlebotomus papatasi* (**B**) were separated on 10% SDS–PAGE gel and incubated with dog sera as follows. **A** Sera selected for the *P. tobbi* immunoprecipitation assay: 1 = a pool of the three sera highly positive for anti-*P. tobbi* saliva IgG, 2 = a pool of the three sera negative for anti-*P. tobbi* saliva IgG, 3–5 = reactivity of the individual sera from the positive pool. **B** Five individual sera (1–5) highly positive for anti-*P. papatasi* saliva IgG. Two main protein bands were subjected to proteomic analysis. Best match representing over 80% of the sample content is indicated on the right side with GenBank accession number in parentheses. Molecular weight (kDa) is indicated on the basis of the protein standards. *SP* salivary protein, *YRP* yellow-related protein

Main salivary antigens in *P. tobbi* and *P. papatasi* saliva were identified by immunoprecipitation assay. Three highly positive dog sera, selected on the basis of their reactivity in ELISA and immunoblot (Fig. 2), were subjected to the immunoprecipitation assay with salivary gland homogenate of the respective sand fly species as an antigen. The eluted antigens were analysed by mass spectrometry (Tables 1 and 2), which revealed several candidate proteins enriched in the sample with pooled positive sera, out of which five were selected for recombinant production (TOB-SP10, TOB-SP38, TOB-SP56, TOB-SP60, and PAP-SP40; Table 3). In the case of TOB-SP60, the selected candidate protein was not the best match but showed better bioinformatic characteristics such as the absence of the putative glycosylation sites (Table 3). Although the other three *P. papatasi* proteins (PAP-SP32, PAP-SP36 and PAP-SP42) did not show promising features in the immunoprecipitation assay, they have been included among candidates as being abundant in positive bands on immunoblot (PAP-SP36 and PAP-SP42; Fig. 2B and Table 3) or in the case of PAP-SP32 as an already well-studied marker of sand fly exposure [51, 52].

**Table 1:** Immunocapture proteomic analysis of *Phlebotomus tobbi* salivary antigens

Best match GenBank accn. no	Protein description	Positive sera signal intensity	Negative sera signal intensity	Unique peptides	Unique sequence coverage (%)	Molecular weight (kDa)
ADJ54103	24.5 kDa SP	24.100	25.495	1	9.0	26.6
ADJ54102	24.53 kDa SP	25.968	25.881	1	9.0	26.6
ADJ54100	28.0 kDa SP	23.979	NaN	1	5.7	30.3
ADJ54098	38.8 kDa SP	26.779	26.529	5	11.5	41.1
ADJ54083	28.8 kDa antigen 5-related SP	26.171	27.233	6	36.3	24.8
ADJ54078	35.7 kDa salivary apyrase	24.761	NaN	4	14.5	37.7
ADJ54077	35.2 kDa salivary apyrase	27.565	24.890	9	34.0	37.4
ADJ54092	25.9 kDa D7-related SP	25.229	NaN	1	5.8	27.8
ADJ54090	25.3 kDa D7-related SP	21.087	NaN	1	4.7	27.3
ADJ54094	26.96 kDa D7-related SP	26.973	NaN	3	19.0	29.0
ADJ54080	42.6 kDa yellow-related SP	22.427	NaN	3	12.7	44.5

Sera signal intensity values refer to the signal intensity obtained from a protein sample derived from a salivary gland homogenate that has been incubated with sera either positive or negative for anti-*P. tobbi* SGH IgG. *SP* salivary protein, *accn. no.* accession number, *NaN* not a number (i.e. no reactivity is expected as the intensity signal was not obtained).

**Table 2:** Immunocapture proteomic analysis of *Phlebotomus papatasi* salivary antigens

Best match GenBank accn. no	Protein description	Positive sera signal intensity	Negative sera signal intensity	Unique peptides	Unique sequence coverage (%)	Molecular weight (kDa)
XP_055702293	Apyrase-like	23.408	NaN	2	6.0	38.2
AAG17637	SP36 apyrase	26.259	23.557	0	0.0	38.2
XP_055703498	SP34 lufaxin-like	21.261	NaN	16	54.4	36.3
AGE83083	SP12	22.007	NaN	2	7.9	16.0
AEH76103	SP15	26.263	24.274	0	0.0	14.5
AEH76113	SP15	25.932	24.606	0	0.0	14.4
QMU26422	SP15, partial	21.680	NaN	1	6.3	16.6
AEH76099	SP15, partial	25.621	NaN	0	0.0	14.6
XP_055709290	SP28, partial	21.821	23.387	6	11.8	29.2
AGE83096	SP29	25.236	22.342	0	0.0	30.9
XP_055709291	SP30	26.493	22.161	16	49.0	29.9
QCZ41610	SP32	25.759	23.170	2	17.5	20.7
AAL11051	SP42	26.309	23.344	0	0.0	44.5
QCZ41862	SP42, partial	23.826	22.467	0	0.0	23.0
ARE29947	SP42-like protein	23.506	22.316	0	0.0	34.8
ARE29946	SP42-like protein	22.801	NaN	5	11.9	34.7
AGE83095	SP44	23.327	24.351	4	7.0	45.7
XP_055705662	SP40	28.718	NaN	3	13.8	42.3

Sera signal intensity values refer to the signal intensity obtained from a protein sample derived from a salivary gland homogenate that has been incubated with sera either positive or negative for anti-*P. papatasi* SGH IgG. *SP* salivary protein, *accn. no.* accession number, *NaN* not a number (i.e. no reactivity is expected as the intensity signal was not obtained).

**Table 3:** A list of selected candidate proteins

Protein name	GenBank accn. no	Protein family	Molecular weight	Best match	Putative N-glycosylation	Selection criteria
TOB-SP10	ADJ54078	Apyrase	37.706	Yes	None	IP
TOB-SP38	ADJ54080	Yellow-related protein	44.515	Yes	1	IP
TOB-SP56	ADJ54092	D7-related	27.768	Yes	None	IP
TOB-SP60	ADJ54095	D7-related	28.979	No	None	IP
PAP-SP32	AFY13225.1	SP32	20.663	N.A	None	literature
PAP-SP36	AAG17637.1	Apyrase	38.157	Yes	2	IB
PAP-SP40	XP_055705662	Serine protease-like	42.337	Yes	1	IP
PAP-SP42	AAL11051.1	Yellow-related protein	44.459	Yes	None	IB

TTT*OTOB* recombinant protein derived from *Phlebotomus tobbi* saliva; *PAP* recombinant protein derived from *P. papatasi* saliva. Molecular weight was calculated from the protein spectra. Best match indicates whether the protein is the best match obtained in proteomic analysis. *Putative N-glycosylation* in the amino acid sequence of the protein without the signal peptide. *IP* immunoprecipitation assay, *IB* immunoblot, *N.A.* not applicable, *SP* salivary protein, *accn. no.* accession number.

### Evaluation of recombinant protein candidates

**Fig. 3:**
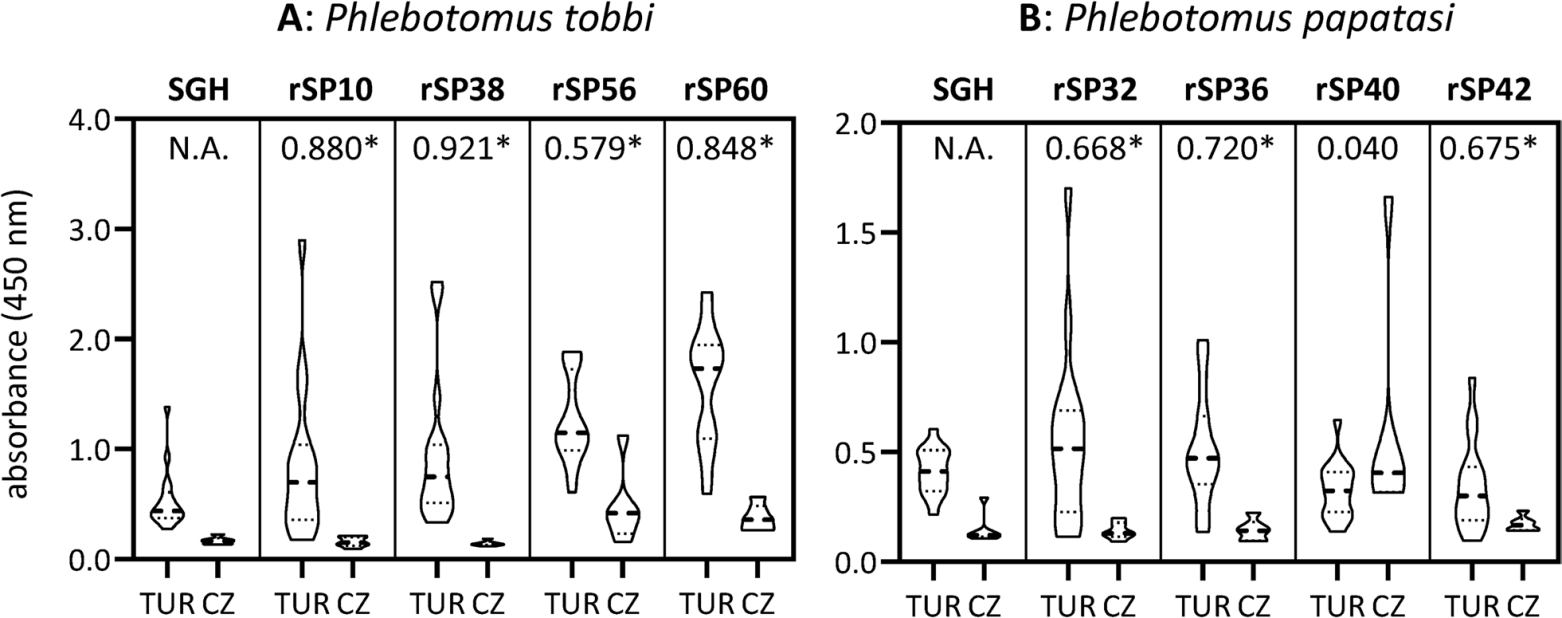
Evaluation of the recombinant protein candidates in small-scale ELISA test. Sera were collected in Türkiye (TUR; *n* = 16) in *Phlebotomus tobbi*- or *P. papatasi*-endemic areas or in the Czech Republic (CZ; *n* = 6), the sand fly-free area, and subjected to ELISA test with antigen, as indicated above the graph frame, to detect specific IgG antibodies. The data are presented as a violin plot, with the dashed line marking the median value and the two dotted lines representing Q1 and Q3 quartiles. Asterisks (*) indicate significant correlation coefficient (*P* < 0.05) with salivary gland homogenate of the respective species by using non-parametric Spearman correlation test

The eight bacterially expressed proteins (TOB-rSP10, TOB-rSP38, TOB-rSP56, TOB-rSP60, PAP-rSP32, PAP-rSP36, PAP-rSP40, and PAP-rSP42) were tested in ELISAs with 22 selected dog sera showing the full range of anti-*P. tobbi* or anti-*P. papatasi* SGH IgG levels, respectively. The results were correlated with the reactivity of the same panel of sera tested on SGH as an antigen (Fig. 3).

Among *P. tobbi* recombinants, TOB-rSP38 showed the highest positive correlation with SGH (*r* = 0.921, *P* < 0.001), followed by very good correlation of TOB-rSP10 and TOB-rSP60 (*r* = 0.880 and 0.848, respectively, *P* < 0.001), and moderate correlation of TOB-rSP56 (*r* = 0.579, *P* = 0.005).

Among *P. papatasi* recombinants, all proteins showed substantial correlation with *P. papatasi* SGH (*r* = 0.668–0.720, *P* < 0.001) except for PAP-rSP40, which did not significantly correlate with SGH (*r* = 0.040, *P* = 0.86). The latter one was thus excluded from further experiments.

### Validation of recombinant protein candidates

**Fig. 4:**
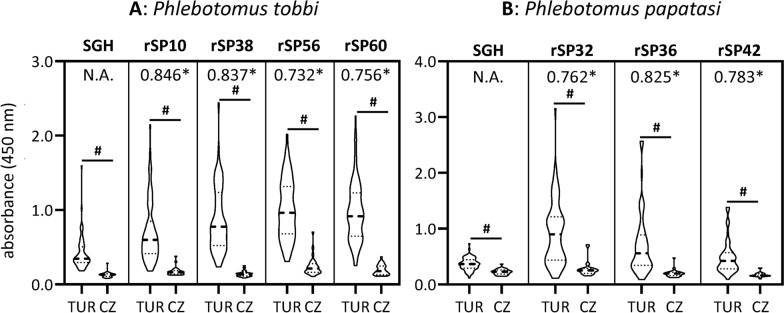
Validation of the recombinant protein candidates in large-scale ELISA test. Sera were collected in Türkiye (TUR; *n* = 64), in *Phlebotomus papatasi*-endemic area, or in the Czech Republic (CZ; *n* = 27), the sand fly-free area, and subjected to ELISA test with antigen, as indicated above the graph frame, to detect specific IgG antibodies. The data are presented as a violin plot, with the dashed line marking the median value and the two dotted lines representing Q1 and Q3 quartiles. Asterisks (*) indicate significant correlation coefficient (*P* < 0.01) with salivary gland homogenate of the respective species by using non-parametric Spearman correlation test. Hashtags (#) indicate significant differences (*P* < 0.01) between Turkish sera and negative controls, using Kruskal–Wallis test with Dunn’s multiple comparison

The seven bacterially expressed proteins (TOB-rSP10, TOB-rSP38, TOB-rSP56, TOB-rSP60, PAP-rSP32, PAP-rSP36, and PAP-rSP42) were further tested in ELISA with 92 selected dog sera showing the full range of anti-*P. tobbi* or anti-*P. papatasi* SGH IgG levels, respectively. The assay variables (cut-off, sensitivity, specificity, PPV and NPV) were calculated on the basis of the reactivity of the same panel of sera with SGH as a gold standard antigen (Fig. 4).

Among *P. tobbi* recombinants, TOB-rSP10 and TOB-rSP38 proved to be the best candidate antigens, again showing very good positive correlation with SGH (*r* > 0.83, *P* < 0.001), followed by substantial correlation of other two TOB recombinants, TOB-rSP56 and TOB-rSP60 (*r* = 0.732 and 0.756, *P* < 0.001) (Table 4).

**Table 4:** Validation of the *Phlebotomus tobbi* recombinant protein candidates

*P. tobbi*	SGH	rSP10	rSP38	rSP56	rSP60
TUR (avg ± SD)	0.420 ± 0.23	0.709 ± 0.42	0.875 ± 0.44	1.012 ± 0.40	0.978 ± 0.41
CZ (avg ± SD)	0.131 ± 0.04	0.174 ± 0.06	0.141 ± 0.04	0.244 ± 0.13	0.196 ± 0.07
Cut-off (avg + 3^*^ SD)	0.247	0.344	0.254	0.625	0.409
No. positive TUR	55	54	63	52	61
No. negative TUR	9	10	1	12	3
No. positive CZ	1	1	1	1	0
No. negative CZ	25	25	25	25	26
Sensitivity (%)		90.9	100	85.5	98.2
Specificity (%)		88.2	73.5	82.4	79.4
PPV (%)		92.6	85.9	88.7	88.5
NPV (%)		85.7	100	77.8	96.4
Spearman *r*		0.846^*^	0.837^*^	0.732^*^	0.756^*^

Sera were collected in *P. tobbi*-endemic areas (Türkiye) or in the sand fly-free area (the Czech Republic), and subjected to ELISA test with antigen, as indicated, to detect specific IgG antibodies.

^*^P < 0.001

*ELISA* enzyme-linked immunosorbent assay, *IgG* immunoglobulin G, *SGH* salivary gland homogenate, *rSP* recombinant salivary protein, *TUR* sera collected in Türkiye, *CZ* sera collected in the Czech Republic, *avg* mean, *SD* standard deviation, *No.* number of positive and negative samples based on the cut-off value, *PPV* positive predictive value, *NPV* negative predictive value, *Spearman r* Spearman correlation coefficient.

Among *P. papatasi* recombinants, PAP-rSP36 proved to be the best candidate antigen, showing very good positive correlation with SGH (*r* = 0.825, *P* < 0.001), followed by substantial correlation of two other PAP recombinants (*r* ranging between 0.762 and 0.783, *P* < 0.001). Besides the best correlation, PAP-rSP36 also showed the best combination of other assay variables (Table 5); the cut-off value was the closest one to the SGH, sensitivity and NPV were around 90%, and specificity (77.4%) and PPV (65.0%) were the highest among the tested proteins.

**Table 5:** Validation of the *Phlebotomus papatasi* recombinant protein candidates

*P. papatasi*	SGH	rSP32	rSP36	rSP42
TUR (avg ± SD)	0.367 ± 0.11	0.930 ± 0.58	0.723 ± 0.57	0.487 ± 0.30
CZ (avg ± SD)	0.226 ± 0.05	0.277 ± 0.13	0.197 ± 0.07	0.176 ± 0.04
Cut-off (avg + 3^*^ SD)	0.377	0.675	0.402	0.303
No. positive TUR	29	38	39	45
No. negative TUR	35	26	25	19
No. positive CZ	0	2	1	0
No. negative CZ	27	25	26	27
Sensitivity (%)		82.8	89.7	93.1
Specificity (%)		74.2	77.4	71.0
PPV (%)		60.0	65.0	60.0
NPV (%)		90.2	94.1	95.7
Spearman *r*		0.762^*^	0.825^*^	0.783^*^

Sera were collected in *P. papatasi*-endemic areas (Türkiye) or in the sand fly-free area (the Czech Republic), and subjected to ELISA test with antigen, as indicated, to detect specific IgG antibodies.

^*^P < 0.001

*ELISA* enzyme-linked immunosorbent assay, *IgG* immunoglobulin G, *SGH* salivary gland homogenate, *rSP* recombinant salivary protein, *TUR* sera collected in Türkiye, *CZ* sera collected in the Czech Republic, *avg* mean, *SD* standard deviation, *No.* number of positive and negative samples based on the cut-off value, *PPV* positive predictive value, *NPV* negative predictive value, *Spearman r* Spearman correlation coefficient.

## Discussion

Several million people and animals worldwide are at risk of contracting SFB pathogens [9, 53]. A key tool to help control SFB diseases is the monitoring of human and/or animal exposure to the vector. For this purpose, anti-sand fly saliva antibodies have been validated as a reliable indicator [13]. Moreover, serological methods are minimally invasive, rather inexpensive and rapid. When combined with entomological screening, such methods enable a more accurate estimate the of risk of SFB pathogen transmission in a given area.

A more feasible alternative to screening the human population would be to monitor domestic animals, which live in close proximity to humans and can therefore act as sentinel hosts. In the case of leishmaniasis caused by *Le. infantum*, the option could be to monitor dogs as the sentinel as well as principal reservoir hosts of this parasite species, the causative agent of CanL and zoonotic human VL. In the Mediterranean area, it is transmitted by sand fly species of the subgenus *Larroussius*, mainly *P. perniciosus* and *P. tobbi*, with allopatric distribution [32–34, 54]. While serological tools to monitor dog exposure to *P. perniciosus* are already available [23–28, 30, 31], there is a lack of equivalent assays for *P. tobbi*. Thus, this study aimed to develop a serological tool on the basis of the recombinant antigen(s) to monitor dog exposure to this important vector.

To select the candidate proteins, we first screened dog sera from a *P. tobbi*-endemic area in Türkiye for anti-saliva antibodies in ELISA by using *P. tobbi* salivary gland homogenate. Dog sera from Türkiye had significantly higher levels of IgG antibodies against *P. tobbi* SGH than the controls from the Czech Republic, the non-endemic country. The Turkish dogs had a wide range of anti-*P. tobbi* saliva antibodies levels. Positive sera showed a consistent pattern on immunoblot, strongly recognizing several antigenic bands. The positive sera were then used to identify main *P. tobbi* salivary antigens by using a novel approach – immunoprecipitation assay.

In previous studies, the main salivary antigens were identified by using electrophoretically separated salivary gland homogenate incubated with sera positive for anti-saliva antibodies, ideally followed by proteomic analysis of the positive band(s) [16, 19, 55–57]. In our study, we tried a different approach; the samples for proteomic analysis were prepared by using the immunoprecipitation assay. The advantage of this method lies in fact that the protein treatment is more similar to an ELISA, having the proteins in their most native form regarding biochemical characteristics. This should ensure that the antibodies will bind to similar epitopes as in an ELISA, thus avoiding the risk arising from potential modification of their antigenic properties during electrophoresis and blotting. Indeed, some sera positive in ELISA failed to react with antigens on immunoblot [58]. Moreover, the protein bands on immunoblot may consist of several different proteins, and the proteomic results can be thus biased if the non-antigenic proteins are more abundant. However, immunoprecipitation assay leads to a sample enriched with proteins that react with serum antibodies, providing a more sensitive approach in identifying salivary antigens with greater accuracy.

Using this approach, we selected four *P. tobbi* candidate proteins, expressed them in *E. coli* and evaluated their reactivity in ELISA with dog sera from endemic areas in Türkiye and a non-endemic area in the Czech Republic. In a small-scale ELISA (22 serum samples), three of four recombinant proteins showed very good correlation above 0.8 when compared with SGH antigen as a gold standard. The large-scale ELISA, comprising more than 90 sera, showed TOB-rSP38 and TOB-rSP10 as the best candidate antigens with correlation still above 0.8. The other two candidates (TOB-rSP56 and TOB-rSP60) also correlated well with SGH, having the coefficient above 0.7. In the receiver operating characteristic (ROC) analysis, area under the curve (AUC) was above 0.98 for all recombinant antigens (Additional File 3: Supplementary Fig. S2), with TOB-rSP38 performing even better than TOB-SGH (0.999 versus 0.991, respectively). When the cut-off value was arbitrarily set as mean of non-endemic controls + 3 standard deviations, the TOB-rSP38 antigen had the lowest cut-off, 100% sensitivity and 100% negative predictive value, meaning that all samples tested negative are truly negative. Positive predictive value was 85.9%, suggesting a low level of false-positive results. TOB-rSP10 had the second lowest cut-off value and the best specificity (88.2%) and PPV (92.6%), suggesting a very low level of false positivity. Negative predictive value reached 85.7%, suggesting low possibility of false-negative results among bitten dogs.

Similar workflow was used for the development of the *P. papatasi* immunoassay. Although this sand fly species is not involved in the epidemiology of CanL, it is a specific vector of *Le. major*, which primarily infects rodent hosts and causes zoonotic human CL [32–34]. However, *P. papatasi* is an opportunistic blood feeder, and dogs can thus serve as sentinel hosts. Indeed, dog blood has been found in trapped *P. papatasi* females [11, 36], and naturally bitten dogs have been shown to develop anti-*P. papatasi* saliva antibodies [26]. In our study, Turkish dogs exhibited significantly higher levels of IgG antibodies against *P. papatasi* SGH than the controls from the non-endemic country. Positive sera were subsequently used to identify the main salivary antigens by a combination of immunoprecipitation and immunoblotting assays.

Out of the four recombinant candidate *P. papatasi* proteins, only three showed significant correlation with SGH in a small-scale ELISA and were thus included in further analysis. In a large-scale ELISA, correlation was stronger for all three recombinants, with PAP-rSP36 correlated as the best (*r* = 0.825) and the other two having very good correlation as well (above 0.75). In the ROC analysis, AUC values for all three recombinant antigens were above 0.9, higher than for PAP-SGH (AUC = 0.882) (Additional File 3: Supplementary Fig. S2). Using the same calculation for the cut-off value as in *P. tobbi* ELISA, PAP-rSP36 and PAP-rSP42 had the lowest cut-off and the highest sensitivity (89.7% and 93.1%, respectively). PAP-rSP36 also showed the best specificity (77.4%) and PPV (65%). Although both parameters were the best among the three tested recombinants, their values are not optimal and suggest possible false positivity among non-bitten dogs. Based on the immunoprecipitation assay, there are several other candidates that are worth testing as markers of exposure in future studies, e.g. SP12, SP15, lufaxin-like protein (GenBank accn. no. AGE83083, QMU26422, XP_055703498), or other apyrase and SP42 yellow-related protein (GenBank accn. no. XP_055702293 and ARE29954, respectively).

The eight candidate proteins tested in our study belong to five different protein families – apyrases (TOB-rSP10 and PAP-rSP36), yellow-related proteins (TOB-rSP38 and PAP-rSP42), SP32-like proteins (PAP-rSP32), D7-related proteins (TOB-rSP56 and TOB-rSP60) and serine proteases (PAP-rSP40).

Apyrases are enzymes that catalyse the hydrolysis of extracellular ATP and ADP, thus neutralizing the host signal initiating platelet aggregation. Several recombinant sand fly apyrases have already been evaluated as markers of exposure with dog sera [16, 19, 21, 22]. In *L. longipalpis*, LJL23 apyrase (GenBank accn. no. AF131933) reacted with canine anti-*L. longipalpis* saliva antibodies on immunoblot [16] but has never been evaluated in ELISA. In *P. orientalis*, PorSP15 apyrase (GenBank accn. no. AGT96431) reacted in ELISA with positive sera from naturally bitten Ethiopian dogs, correlating well with SGH (*r* = 0.726, *n* = 36), nevertheless showing mild sensitivity (68%) and low specificity (58.8%, albeit the highest one among the tested recombinants) [19]. Two apyrases from *P. perniciosus* have been evaluated by ELISA with dog sera as well, rSP01 and rSP01B (GenBank accn. no. KF257365 and KF257364, respectively). Both correlated well with SGH when using sera of dogs experimentally bitten by *P. perniciosus* (*r* = 0.89–0.91, *n* = 12) [21] and of naturally bitten Spanish dogs (*r* = 0.86, *n* = 34) [22]. In our study, both *P. tobbi* and *P. papatasi* recombinant apyrases correlated the best with SGH among the other tested recombinants (*r* = 0.846 and 0.825, respectively). They also performed well regarding sensitivity (around 90%), with a relatively low level of false negativity (specificity being 88.2% and 77.4%, respectively), proving to be promising markers of sand fly exposure.

Yellow-related proteins (YRPs) in sand fly saliva are scavengers of host bioamines (such as serotonin or histamine), the damage-associated molecules that trigger the haemostatic reaction [59–61]. They are also reliable markers of exposure in dogs, as demonstrated for *L. longipalpis* LJM11, LJM17 and LJM111 (GenBank accn. no. AY445935, AF132518 and DQ192488, respectively) [16, 18]; *P. orientalis* PorSP24 (GenBank accn. no. AGT96428) [19]; and *P. perniciosus* SP03B (GenBank accn. no. DQ150622) [21, 22]. *Lutzomyia longipalpis* LJM111 has been tested only on immunoblot [16], but the combination of LJM11 and LJM17 has been recently evaluated also in ELISA with sera of naturally bitten Brazilian dogs (*n* = 177), correlating well with SGH (*r* = 0.91) and showing high sensitivity (81%) and specificity (92%) [18]. *Phlebotomus orientalis* PorSP24 also correlated quite well with SGH (*r* = 0.790, naturally bitten Ethiopian dogs, *n* = 36), providing 100% sensitivity and NPV combined with moderate specificity (41.2%) and PPV (65.5%) [19], thus indicating relatively high probability of false-positive samples. *Phlebotomus perniciosus* SP03B correlated well with SGH (*r* = 0.83–0.89) in evaluation studies with low number of dog sera [21, 22, 29]. During the follow-up validation steps with higher number of sera from naturally bitten dogs, correlation with SGH ranged between 0.48 and 0.82, with *r* = 0.7 as the median value [23–28, 31]. In our study, both *P. tobbi* and *P. papatasi* recombinant YRPs correlated well with SGH (*r* = 0.836 and 0.783, respectively) in both cases as the second-best candidates after the apyrases. They proved to have the highest AUC value (0.999 and 0.943, respectively) and the highest sensitivity (100% and 93.1%, respectively), but the worst specificity (73.5% and 71%, respectively) and PPV (85.9% and 71%, respectively) among the tested candidate recombinants, suggesting that the rYRP-based immunoassays may detect all positive samples but with a higher risk of false positivity among non-bitten dogs. Since *P. orientalis* YRP performed similarly in these parameters [19], the high sensitivity and low specificity might be a common feature of recombinant YRPs; however, this needs to be confirmed with rYRPs from other *Phlebotomus* species.

Since the sensitivity is highest in YRPs and the specificity in apyrases, the combination of these two recombinants from the respective species may theoretically improve the immunoassay performance. However, in *P. perniciosus*, such a combination (rSP03B YRP with rSP01 apyrase) showed contradictory results regarding the assay improvement compared with rSP03B alone. On the basis of the correlation coefficient, it showed no effect in an Italian study (*r* = 0.65 versus 0.77, *n* = 56; [23]), contrary to slight improvement in a study on Portuguese dogs (*r* = 0.841 versus 0.820, *n* = 208; [27]). Nevertheless, sensitivity and specificity calculations have not been provided in any of these studies.

The SP32-like proteins are sand fly-specific, predicted to have mucin-like properties, but their function remains unknown [62]. Their recombinant counterparts have been found to be valid alternatives to SGH in human immunoassays measuring exposure to *P. papatasi* [51, 52] and *P. argentipes* [57]. To our knowledge, there is only one study assessing a member of this protein family in canine ELISA, showing only moderate correlation with SGH (*r* = 0.46, *n* = 32) [26]. The newly expressed PAP-SP32 recombinant used in this study correlated well with SGH (*r* = 0.762, *n* = 91), with the lowest sensitivity (82.8%), similar specificity (74.2%) and highest cut-off value [optical density (OD) = 0.675] among the other tested *P. papatasi* recombinants. Although the assay parameters were not the best, they were comparable or better than the human immunoassay based on *P. papatasi* SP32 recombinant protein that showed only moderate correlation with SGH (*r* = 0.544, *n* = 522), sensitivity of 80.4% and specificity of 71.6% [52].

D7 proteins in sand fly saliva have two domain structures and are able to bind cysteinyl leukotrienes and thromboxane A_2_, thus serving as anti-inflammatory and anti-platelet agents [63]. Recombinant D7 protein from *L. longipapis* LJL13 (GenBank accn. no. AF420274) has been found to bind dog anti-saliva antibodies on immunoblot [16] but has never been evaluated in ELISA. In *P. orientalis*, recombinant D7 protein PorSP67 (GenBank accn. no. AGT96467) correlated well with SGH in ELISA with dog sera (*r* = 0.687, *n* = 13), but with the worst performance among the four tested candidates in the follow-up ELISA [19]. *Phlebotomus perniciosus* recombinant D7 protein SP04 (GenBank accn. no. KF178456) did not significantly correlate with SGH (*r* = −0.59, *n* = 18) nor react on immunoblot with sera from experimentally bitten dogs [21]. However, *P. tobbi* D7 recombinant proteins TOB-rSP56 and TOB-rSP60 evaluated in this study correlated well with SGH (*r* > 0.73), also showing high AUC values in the ROC analysis (> 0.98). The sensitivity (85.5% and 98.2%, respectively) and specificity (82.4% and 79.4%, respectively) values were between TOB-rSP38 yellow-related protein and TOB-rSP10 apyrase, suggesting overall good performance as markers of exposure.

The last recombinant protein evaluated in this study had the best match with venom serine protease 34-like from the *P. papatasi* genome (GenBank accn. no. XP_055705662). Although this protein has not been found in sand fly sialo transcriptomic studies published so far, we decided to express it and evaluate it in ELISA as a candidate marker of exposure. The rationale behind this decision was the convincing results from the immunoprecipitation analysis, its putative secretory nature (the sequence possesses a signal peptide) and the presence of serine proteases in mosquito saliva [64] or in the venom of bees and wasps [65], suggesting it might be secreted and present in sand fly saliva as well. Since the sequence had predicted an active proteolytic site (H172–D222–S316), it was mutated (S316A) to avoid interference of its proteolytic function with the expression and/or immunoassay performance. Nevertheless, the recombinant protein did not pass the ELISA evaluation step, since it was the only candidate with no significant correlation to SGH (*r* = 0.040) and was thus excluded from further analysis. Ongoing experiments should answer the questions of whether this protein is a part of sand fly saliva and possesses the predicted enzymatic activity, and whether the mutation was effective in its recombinant counterpart.

## Conclusions

Sensitive and accurate tools to measure and monitor SFB pathogen transmission are essential for SFB disease control. Trapping methods using Centers for Disease Control and Prevention (CDC) light traps close to human dwellings or animal shelters can only provide crude population-level estimates of vector exposure that do not necessarily reflect the individual differences in exposure pattern given, e.g. by host species, sex or age. Serological tools based on the measurement of anti-vector saliva antibodies may fill this gap and serve as proxy biomarkers for vector exposure, both on population and individual levels, thus providing key information to assess SFB pathogen transmission risk and consequently guide more targeted vector control interventions. Within the CLIMOS project [66], they will be used to monitor exposure in sentinel dog populations in areas endemic for *Le. infantum*, complementing other surveillance and leishmaniasis control tools to provide the most accurate warning of the circulating *Leishmania* infection.

## Supplementary Information

Additional file 1.

Additional file 2.

Additional file 3.

## Data Availability

The data supporting the conclusions of this article are included within the article.

## Abbreviations

Accn. no.: Accession number
CanL: Canine leishmaniasis
CL: Cutaneous leishmaniasis
CZ: Czech Republic
ELISA: Enzyme-linked immunosorbent assay
IgG: Immunoglobulin G
*L.*: *Lutzomyia*
*Le.*: *Leishmania*
MCL: Mucocutaneous leishmaniasis
N.A.: Not applicable
NPV: Negative predictive value
*P.*: *Phlebotomus*
PAP: *Phlebotomus* *papatasi*
PPV: Positive predictive value
rSP: Recombinant salivary protein
RT: Room temperature
SFB: Sand fly-borne
SGH: Salivary gland homogenate
SP: Salivary protein
TOB: *Phlebotomus tobbi*
TUR: Türkiye
VL: Visceral leishmaniasis
YRP: Yellow-related protein
