# Supplementary material for: Development of new screening tools to evaluate dog exposure to Phlebotomus tobbi and Phlebotomus papatasi sand flies
Source: Parasit Vectors. 2026 Feb 27;19:147. doi: 10.1186/s13071-026-07286-4 (PMC13049789; doi:10.1186/s13071-026-07286-4)
Supplement: Supplementary file 1 — Additional file 1. [file 13071_2026_7286_MOESM1_ESM.docx]

**Supplementary information - Additional File 1**

**Table S1** A list of recombinant proteins produced within this study.

| Recombinant protein name | GenBank accession number | | Protein family | Signal Sequence | Recombinant protein  solubility |
| --- | --- | --- | --- | --- | --- |
|  | Original AA | Original nn |  |  |  |
| TOB-rSP10 | ADJ54078 | HM135952 | apyrase | GEA-APR | yes |
| TOB-rSP38 | ADJ54080 | HM140619 | yellow-related protein | VVG-FHV | no |
| TOB-rSP56 | ADJ54092 | HM164147 | D7-related | GYS-WQY | no |
| TOB-rSP60 | ADJ54095 | HM164150 | D7-related | GFS-WKY | no |
| PAP-rSP32-His | AFY13225 | JX411944 | SP32 | SSS-AST | yes |
| PAP-rSP36 | AAG17637 | AF261768 | apyrase | SEG-APR | no |
| PAP-rSP40 | XP_055705662 | XM_055849687 | serine protease-like | VAA-QTG | no |
| PAP-rSP42 | AAL11051 | AF335491 | yellow-related protein | ALS-DDV | no |

*TOB* recombinant protein derived from *Phlebotomus tobbi* saliva, *PAP* recombinant protein derived from *P. papatasi* saliva, *rSP* recombinant salivary protein, *AA* aminoacid sequence, *nn* nucleotide sequence.
