# Supplementary material for: Development of new screening tools to evaluate dog exposure to Phlebotomus tobbi and Phlebotomus papatasi sand flies
Source: Parasit Vectors. 2026 Feb 27;19:147. doi: 10.1186/s13071-026-07286-4 (PMC13049789; doi:10.1186/s13071-026-07286-4)
Supplement: Supplementary file 2 — Additional file 2. [file 13071_2026_7286_MOESM2_ESM.docx]

**Supplementary information – Additional File 2**

**
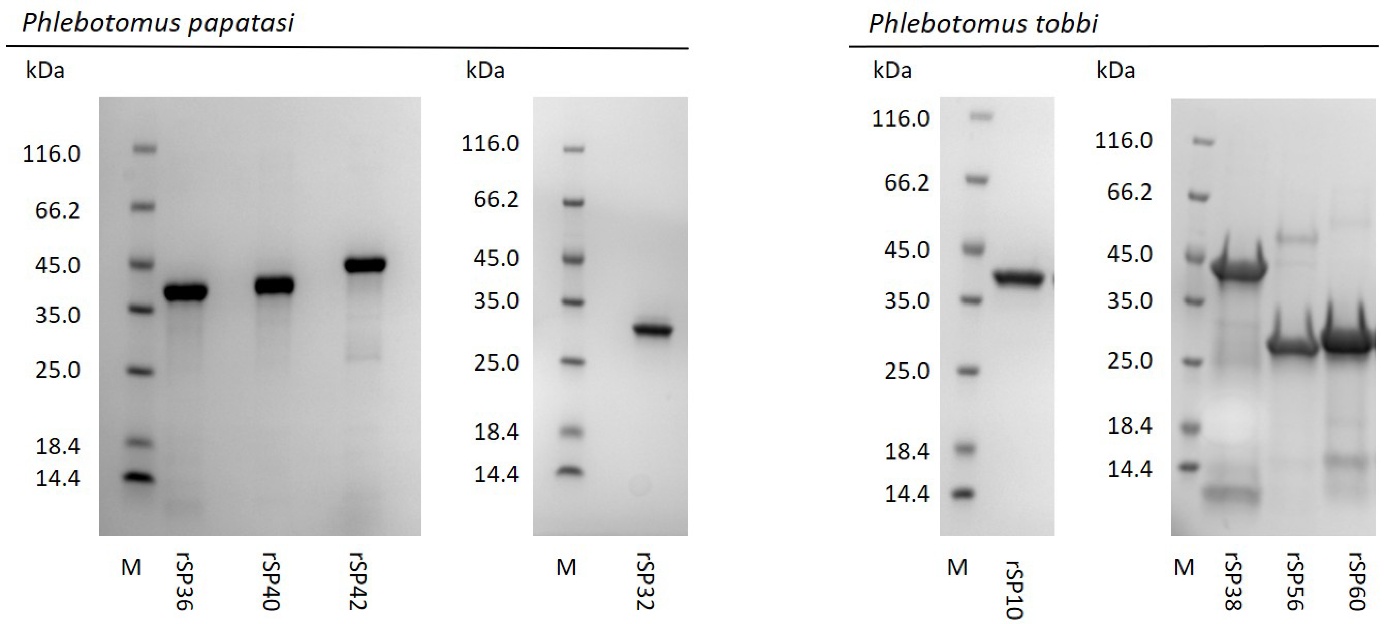
**

**Fig. S1.** SDS-PAGE of recombinant proteins demonstrating their purity level. After the purification step, the sample was loaded onto an 8–16% SDS-PAGE gradient gel (Mini-PROTEAN® TGX™ Precast Gels, Bio-Rad, #4561106) and run at 180 V for approximately 35 minutes. House-made Coomassie staining solution was used to visualise the proteins in the gel (100 mg/L Coomassie Brilliant Blue G-250, 100 mg/L Coomassie Brilliant Blue R-250, 0.3% acetic acid). M = protein marker (peqGOLD Marker I, VWR, #27-1010).
