# Supplementary material for: Development of new screening tools to evaluate dog exposure to Phlebotomus tobbi and Phlebotomus papatasi sand flies
Source: Parasit Vectors. 2026 Feb 27;19:147. doi: 10.1186/s13071-026-07286-4 (PMC13049789; doi:10.1186/s13071-026-07286-4)
Supplement: Supplementary file 3 — Additional file 3. [file 13071_2026_7286_MOESM3_ESM.docx]

**Supplementary information – Additional File 3**

**
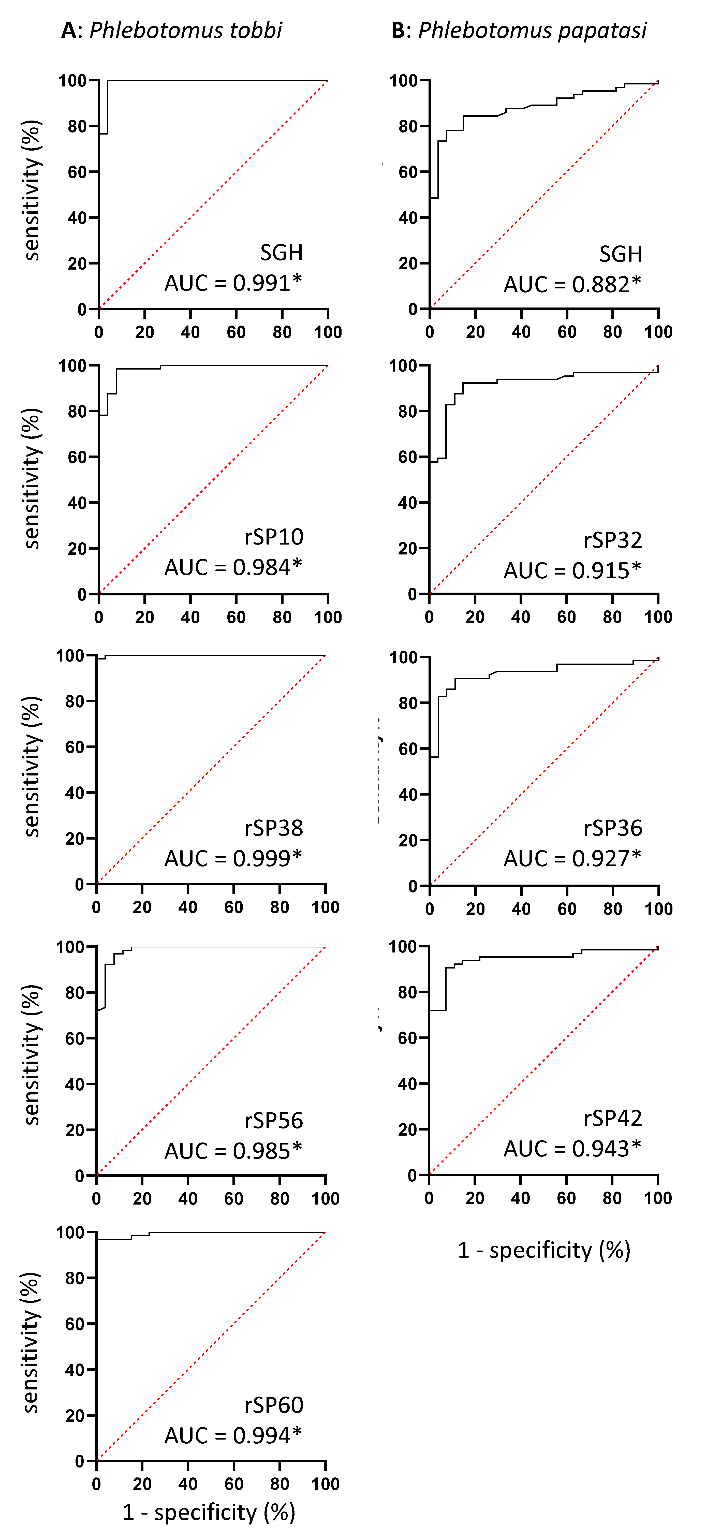
**

**Fig. S2.** ROC curve analysis of the recombinant protein candidates in large-scale ELISA test. Sera were collected in *Phlebotomus papatasi*- or *Phlebotomus tobbi*-endemic areas in Türkiye (TUR, n = 64 per sand fly species), and in the sand fly-free area in the Czech Republic (CZ, n = 27). The same set of sera as in Figure 4 and Tables 4 and 5 were analysed. Antigen used in the ELISA test is indicated in each graph, AUC: area under the curve, * indicate p value < 0.05.
